# Floral volatiles interfere with plant attraction of parasitoids: ontogeny-dependent infochemical dynamics in *Brassica rapa*

**DOI:** 10.1186/s12898-015-0047-7

**Published:** 2015-06-02

**Authors:** Gaylord A Desurmont, Diane Laplanche, Florian P Schiestl, Ted C J Turlings

**Affiliations:** Institute of Biology, University of Neuchâtel, Rue Emile-argand 11, 2000 Neuchâtel, Switzerland; Institute of Systematic Botany, Zollikerstrasse 107, 8008 Zurich, Switzerland

**Keywords:** Plant ontogeny, Herbivore-induced plant volatiles, Indirect defense, Host location, Plant signaling, Floral VOCs

## Abstract

**Background:**

The role of plant ontogeny on investment in direct defense against herbivores is well accepted, but the transition from the vegetative to the reproductive stage can also affect indirect resistance traits (i.e. attraction of the natural enemies of plant attackers). Here, we conducted behavioral bioassays in olfactometers to determine whether the developmental stage (vegetative, pre-flowering, and flowering) of *Brassica rapa* plants affects attraction of *Cotesiaglomerata*, a parasitoid of the herbivore *Pieris brassicae*, and examined the blends of volatile compounds emitted by plants at each developmental stage.

**Results:**

*Pieris*-infested plants were always more attractive to parasitoids than control plants and plants infested by a non-host herbivore, independently of plant developmental stage. On the other hand, the relative attractiveness of *Pieris*-infested plants was ontogeny dependent: *Pieris*-infested plants were more attractive at the pre-flowering stage than at the vegetative stage, and more attractive at the vegetative stage than at the flowering stage. Chemical analyses revealed that the induction of leaf volatiles after herbivory is strongly reduced in flowering plants. The addition of synthetic floral volatiles to infested vegetative plants decreased their attractiveness to parasitoids, suggesting a trade-off between signaling to pollinators and parasitoids.

**Conclusion:**

Our results show that putative indirect resistance traits are affected by plant development, and are reduced during *B. rapa* reproductive stage. The effects of ontogenetic shifts in resource allocation on the behavior of members of the third trophic level may have important implications for the evolution of plant defense strategies against herbivores.

**Electronic supplementary material:**

The online version of this article (doi:10.1186/s12898-015-0047-7) contains supplementary material, which is available to authorized users.

## Background

The production of secondary metabolites (i.e. compounds that are not directly involved in the growth or reproduction of the plant) detrimental to herbivores is one of the main strategies plants have evolved to fend off their consumers. Secondary metabolites can directly deter or negatively affect the performance of herbivores (direct defense) [1], or attract and facilitate the action of natural enemies of herbivores (indirect resistance) [2]. Investment in secondary metabolism can vary tremendously through the lifetime of single plants [3]. Such temporal changes can be separated in two categories: changes driven by fluctuating environmental conditions are referred to as seasonal, and changes associated with the development of the plant are referred to as ontogenetic [4]. Both types of temporal variation can play an important role in plant defense and insect–plant interactions. For example, decrease in plant resistance due to suboptimal environmental conditions may create a window of vulnerability to herbivores [5]. Additionally, plants may not have the same needs for defenses or equal amounts of resources to invest in defensive compounds at all points of their development [3], and such variation may also lead to herbivore adaptation (e.g. herbivore adjusting its “offense” to ontogenetic shifts in plant defense) [6, 7].

The role of ontogeny on direct defenses has been thoroughly studied in the context of the general trade-off between defense and growth/reproduction in plants [8, 9]. It is generally admitted that plant tissues are more valuable, and herbivory more impactful, at the early stages of development [10], and thus that young plant tissues (e.g. seedlings and saplings for herbaceous plants, young expanding leaves for trees) should be more protected than older ones [11], as predicted by the optimal defense hypothesis [12, 13]. However, plants may not always have the capacity to protect young tissues optimally: resource limitations may constrain the production of defensive compounds until the plant has gained enough biomass to allocate resources for defenses [14]. As plants mature and reach the reproductive stage, the dynamics of defenses may change again: levels of defensive compounds may be reallocated to valuable reproductive tissues (e.g. flowers, seeds), or globally decrease if the production of secondary metabolites constrains the production of reproductive tissues [3, 15]. In the case of insect-pollinated plants, the production of defensive compounds may also diminish if those interfere with the action of mutualist pollinators [16]. In contrast to these studies on ontogeny-mediated variation in direct defenses, much less experimental work exists on the effects of plant development on indirect resistance.

Indirect resistance refers to plant adaptations encouraging the presence of natural enemies of insect herbivores, which in turn help plants by reducing herbivore damage. When the action of natural enemies consistently results in increased plant fitness, the term “indirect defense” may be used [2]. These adaptations fall into two main categories: rewards such as food or shelters for natural enemies (e.g. extrafloral nectaries, domatia) [17], and cues facilitating foraging behavior (e.g. herbivore-induced volatiles) [18, 19]. These adaptations have the potential to be ontogeny-dependent: organs rewarding natural enemies may not be developed in the early developmental stages [20], or the emission of plant volatiles may fluctuate over the plant’s lifetime [21].

Several studies have shown that blends of herbivore-induced plant volatiles (HIPVs) can vary over a plant’s lifetime [22], but how these ontogeny-driven changes in HIPVs affect the foraging behavior of natural enemies is vastly unknown [23], particularly during the transition between the vegetative stage and the reproductive stage [24, 25]. This transition is important for members of the third trophic level, because nectar and/or pollen are important food sources for numerous species of natural enemies, including parasitic wasps [26, 27]. Infested flowering plants may offer a “double reward” (host + food) to visiting parasitoids [28], and thus be more attractive than infested vegetative plants. Alternatively, floral volatiles may also interfere with attractive HIPVs in the headspace and reduce their attractiveness to parasitoids. The reliability of HIPVs as specific cues for natural enemies in search of host or prey has been the subject of much debate [29, 30], and ontogeny-driven variations in HIPVs may alter the quality and specificity of the cues natural enemies are looking for, which could have major consequences for the foraging behavior of specialized natural enemies such as parasitoids. Here, we use the plant *Brassica rapa* and one of its major herbivores, the butterfly *Pieris brassicae*, to test whether the attractiveness of herbivore-damaged plants to a specialized natural enemy of *P. brassicae*, the parasitoid *Cotesia glomerata*, changes through plant ontogeny. *Cotesia glomerata* is the main larval parasitoid of *P. brassicae* in Western Europe and can be very abundant in the field [31], but it remains unclear whether *Brassica* plants benefit from its attraction in terms of reduction of herbivore damage and fitness gain [32, 33]. Indeed *C. glomerata* kills its host at the end of its larval development [34], once most of the damage has been done to the plant. In order to test the specificity of the volatiles cues emitted by *B. rapa* in response to *P. brassicae*, we compared *C. glomerata* attraction toward *Pieris*-infested plants to plants infested by a non-host herbivore, *Spodoptera littoralis* through plant ontogeny. Specifically, we used behavioral bioassays to answer the following questions:

(1) Are pre-flowering and flowering plants infested by *Pieris* caterpillars attractive to parasitoids, and how does this attractiveness compare to infested vegetative plants and non-infested plants?

(2) Do *C. glomerata* wasps show preferences between volatiles emitted by plants infested by its host compared to volatiles emitted by plants damaged by a non-host herbivore, *S. littoralis*, at different plant developmental stages?

Because we saw a decrease in attractiveness to parasitoids in infested flowering plants, we then used manipulative experiments to test two potential proximal mechanisms explaining this result: (a) floral volatiles directly interfere with attractive leaf volatiles, and (b) a behavioral change from folivory to florivory (i.e. eating flowers) in *P. brassicae* caterpillars leads to reduced parasitoid attraction.

(3) How do herbivore-induced plant volatiles vary through plant ontogeny? In addition to the behavioral assays abovementioned, we conducted chemical analyses to measure quantitative and qualitative variation in volatile emissions between undamaged and *Pieris*-infested plants at each of the developmental stages tested (vegetative, pre-flowering, and flowering)

## Methods

### Insect and plant material

**Figure 1:**
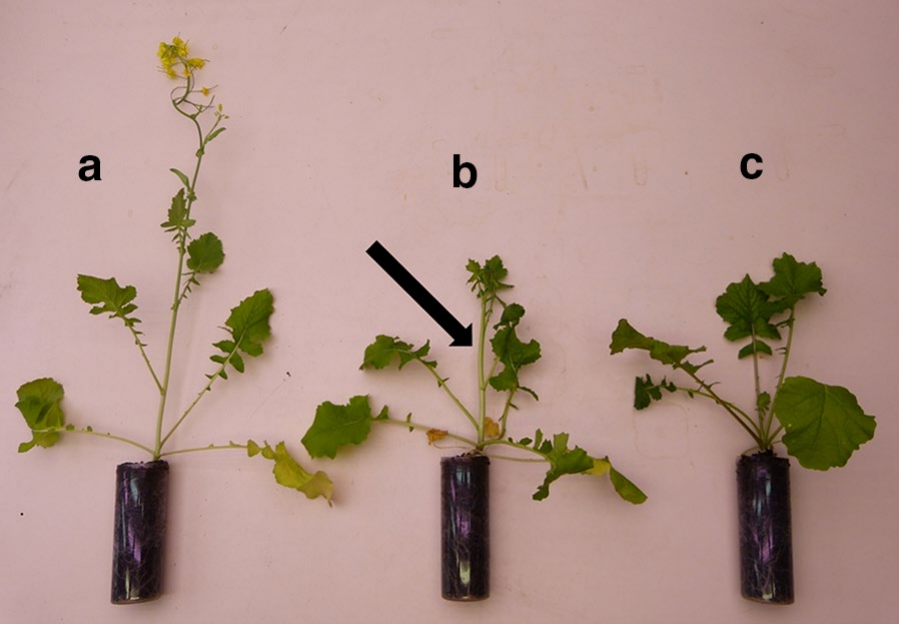
*Brassica rapa* plants at the different developmental stages used in the study: flowering stage (**a**), pre-flowering stage (**b**), and vegetative stage (**c**). The *arrow* indicates the flowering stalk, indicative that the plant has entered the pre-flowering stage.

Plants used in the study came from a wild accession of *B. rapa* whose seeds were collected in 2012 near Maarsen, the Netherlands. Plants were grown in controlled growth chambers under 16:8 L:D light regime at 25°C, in cylindrical plastic pots (4 × 10 cm) with fertilized commercial soil (Ricoter Aussaaterde, Aarberg, Switzerland). Plants were between 3 and 7 weeks old when used for the experiments: plant age could not be standardized because the time at which plants would enter the reproductive stage was unpredictable. Plants were considered in the vegetative stage when they only bore leaves, in the pre-flowering stage once they produced a 8–10 cm long flowering stalk (ca. 5 day before flowering), and in the flowering stage once they started producing open flowers (Figure 1). Plants in the flowering stage were used 2–15 days after the opening of the first flower.

*Pieris brassicae* (Lepidoptera: Pieridae) is a specialist herbivore of crucifers widespread in most of Eurasia. The larvae are typically leaf-feeders, but are known to switch from folivory to florivory after the second larval instar on *Brassica nigra* plants [35], as well as on our *B. rapa* plants (GAD personal observation). *P. brassicae* came from our laboratory rearing, started with field-collected individuals from the Zürich area (Switzerland), and were reared on *Brassica* plants. In order to prepare the *Pieris*-infested plants used for the behavioral bioassays and the chemistry analysis, 15 first instar (L1) larvae were randomly placed on the leaves of the treatment plant using a fine brush the day prior to the experiment. Herbivores were left feeding on the plant during the experiment. First instar larvae were used for infestation treatments because they are the most susceptible to parasitism by *Cotesia glomerata*.

The generalist herbivore *Spodoptera littoralis* is native to Africa and invasive in the southern part of Europe [36]. Individuals of *S. littoralis* used for the experiments came from eggs shipped weekly by Syngenta (Stein, Switzerland). In order to prepare *Spodoptera*-infested plants for the experiments, 25 first and second instar larvae were placed randomly on the leaves of the treatment plant using a fine brush 12–18 h prior to the experiment. Herbivores were left feeding on the plant during the experiment. We used more *S. littoralis* larvae than *P. brassicae* larvae for herbivore-infested treatments (25 vs. 15) because these numbers of larvae resulted in comparable amounts of damage on *B. rapa* leaves after 24 h during preliminary experiments.

The specialist natural enemy of *P. brassicae*, the parasitic wasp *C. glomerata* (Hymenoptera: Braconidae) is a major endoparasitoid of *P. brassicae* in temperate Western Europe, and parasitizes early instars of the caterpillar. Individuals of *C. glomerata* used for the behavioral bioassays came from our laboratory rearing, started with field-collected individuals from the Netherlands and later supplemented with field-collected individuals from the Neuchâtel area (Switzerland). Newly emerged wasps were left in rearing cages at ambient temperature with water and honey for feeding and mating for 48 h, a period that is typically sufficient to ensure successful mating [37]. Then, rearing cages were transferred in a growth chamber at 13°C (16/8 L:D light regime) with water and honey until parasitoids were needed for the experiments (1–6 weeks after emergence). Parasitoid age variability did not differ between the different olfactometer experiments. Parasitoids were removed from their growth chamber and left at ambient temperature for ~1 h before the bioassays. Only naive females (i.e. females that had never encountered a host prior to the experiment) were used in the bioassays, and insects were only used once for experimental purposes.

### Behavioral bioassays

The preferences of *C. glomerata* females toward certain odor blends were investigated using 4 and 6-arm olfactometer settings [38, 39]. In these settings, wasps were given the choices between 4 or 6 odor sources (=treatments) contained in separated glass bottles. Individual air flows were connected to each odor source, and all air flows converged to a central glass piece, where the wasps were released. After 30 min spent in the olfactometer, wasps were recollected and the treatment they chose was recorded. Wasps that did not make a choice were recorded as “no choice” in the analysis of the results. An olfactometer test (=1 replicate) consisted in five consecutive releases of five wasps (wasps were replaced between releases) for the 4-arm olfactometer tests, and in six consecutive releases of six wasps (wasps were replaced between releases) for the 6-arm olfactometer tests. We conducted a minimum of five replicates for each experiment. Plants were changed and glassware was cleaned between replicates. The cleaning process of the glassware consisted in rinsing the glassware sequentially with three solvents: water, acetone, and pentane, and putting the glassware in an oven at 250°C for a minimum of 3 h. A minimum of five replicates were conducted for each of the experiments described below.

### Chemical analysis

To identify and quantify the blends of volatile organic compounds (VOCs) emitted by undamaged and infested *B. rapa* plants at the vegetative and flowering stage, plants (n = 14, 12, and 12 for the vegetative, pre-flowering, and flowering stage, respectively) were placed in a VOC collection setup [40] for 5 h. VOCs were collected using a trapping filter containing 25 mg of 80–100 mesh SuperQ absorbent. Before use, trapping filters were cleaned with 300 μL of methylene chloride. After each collection, VOCs were extracted from the filters with 150 μL of methylene chloride. The collection was performed first with undamaged plants then, using the same plants, 24 h after infestation by 15 L1 *P. brassicae* caterpillars. Caterpillars were not removed from the plants during the volatile collection.

The samples were stored at −80°C before analysis. Two internal standards (*n*-octane and nonyl acetate, each 200 ng in 10 mL methylene chloride) were added to each sample. VOCs were analysed with an Agilent 6850 gas chromatograph with a flame ionization detector. A 2 μL aliquot of each sample was injected in the pulsed splitless mode onto a non-polar column (HP-1 ms, 30 m, 0.25 mm ID, 0.25 μm film thickness, Agilent J&W Scientific, USA). Helium at constant flow (1.9 mL/min) was used as carrier gas. The quantities of the major components of the blends were estimated based on the peak areas of the compounds compared to the peak areas of the internal standards. Identities of the compounds were confirmed by mass spectrometry analysis whenever possible. Compounds were identified by comparing the spectra obtained from the samples with those from a reference database (NIST mass spectral library).

### Experiments

(1) Are pre-flowering and flowering plants infested by *Pieris* caterpillars attractive to parasitoids, and how does this attractiveness compare to infested vegetative plants and non-infested plants?

To determine the attractiveness of *Pieris*-infested plants compared to control plants at each developmental stage, two separated choice-tests, one with vegetative plants and one with flowering plants, were conducted in 4-arm olfactometers (n = 5 replicates, 125 wasps tested). For these tests, wasps were given the choice between a *Pieris*-infested plant, a non-infested plant (control), and two empty odor sources (blanks). Then, to directly compare the attractiveness of *Pieris*-infested vegetative and flowering plants, a 6-arm olfactometer test was conducted, giving the wasps the following choices: a *Pieris*-infested vegetative plant, a *Pieris*-infested flowering plant, a non-infested vegetative plant, a non-infested flowering plant, and two empty odor sources (blanks) (n = 5, 180 wasps tested). The same experiment was repeated with pre-flowering plants instead of flowering plants (n = 5, 180 wasps tested).

(2) Do *C. glomerata* wasps show preferences between volatiles emitted by plants infested by its host compared to volatiles emitted by plants damaged by a non-host herbivore, *Spodoptera littoralis*, at different plant developmental stages?

In order to determine the specificity of HIPVs produced by infested plants at different developmental stages, two separate 4-arm olfactometer choice-tests were conducted, one with vegetative plants and one with flowering plants (n = 5, 125 wasps tested). For these tests, wasps were given the choice between a *Pieris*-infested plant, a *Spodoptera*-infested plant, a non-infested plant, and one empty odor source (blank).

(a) Do floral volatiles directly interfere with attractive HIPVs?

We used synthetic blends of floral volatiles and vegetative *Pieris*-infested plants to determine the influence of floral odors on the foraging behavior of parasitoids. Synthetic blends of volatiles were preferred to real inflorescences for this experiment to limit variability. Specifically we conducted a 4-arm olfactometer experiment, giving the wasps the following choices: *Pieris*-infested vegetative plant, *Pieris*-infested vegetative plant + synthetic floral blend, non-infested vegetative plant, and an empty odor source (blank) (n = 5, 125 wasps tested). Synthetic floral blends included six of the most abundant compounds found in the floral bouquet of our wild accession of *B. rapa* (Knauer and Schiestl unpublished): phenylacetaldehyde (≥90%, Sigma-Aldrich, Buchs, Switzerland) 3 μL/mL, nonanal (Givaudaudan, Dübendorf, Switherland) 9 μL/mL, decanal (Givaudaudan) 4 μL/mL, acetophenone (Givaudaudan) 24.5 μL/mL, *p*-Anisaldehyde (puriss. Sigma Aldrich) 27 μL/mL, and α-Farnesene (mixture of isomers, Sigma Aldrich) 492 μL/mL, diluted in methylene chloride (HPLC grade). Before olfactometer tests, rubber septa (GR-2, 5 mm Supelco, Bellefonte, PA, USA) were soaked in the synthetic floral blend solution for 1 h, then were allowed to dry for 4 h. The concentration of each compound in the solution was adjusted so that the emission rate of each compound from a septa was comparable to one inflorescence (ca. 30 flowers) of *B. rapa* [41] Septa were then placed above the chosen treatment plant just prior to the tests, using a fine wire mesh to fix them at the desired location inside the olfactometer. Fine wire mesh was also added to the treatments without synthetic floral blends in order to account for the potential effects of wire odors. Preliminary trials were conducted before the experiments to show that rubber septa soaked only in solvent (methylene chloride) did not have an effect on parasitoid attraction under the same experimental conditions.

(b) Is the behavioral change from folivory to florivory in *P. brassicae* caterpillars linked to reduced attractiveness to parasitoids?

When they were placed randomly on flowering plants and let free for 24 h, *P. brassicae* first instar larvae generally disperse across the plant and damaged both leaves and flowers. Thus, to test the influence of florivory on parasitoid attraction, we gave the choice to parasitoids between *Pieris*-infested plants with larvae restrained to the leaves, and *Pieris*-infested plants with larvae let free to feed on both leaves and flowers. A 4-arm olfactometer test was conducted, giving the wasps the following choices: *Pieris*-infested flowering plant, *Pieris*-infested flowering plant whose flowers were bagged in a fine-mesh net to prevent larvae from reaching them, non-infested flowering plant, and an empty odor source (blank) (n = 5, 125 wasps tested). In order to avoid the potential effects of bag odors on parasitoid behavior, a fine-mesh net was added to all the treatments and placed next to the plants.

### Statistical analysis

Preferences of *C. glomerata* females were analyzed for each test using a generalized linear model (GLM) fitted by maximum quasi-likelihood estimation according to Turlings et al. [38]. Means were compared using a Chi square test and a multiple comparison Wilcoxon test (α = 0.01, JMP9). The number of wasps choosing the different branches (treatments) of the olfactometer constituted the dependent variable. Treatments were then compared using the all-pairwise Tukey–Kramer HSD procedure (JMP9). Results are presented as percentages in the figures illustrating olfactometer tests for easier comprehension: percentage attractiveness of a given treatment was calculated as the number of wasps that chose that particular treatment divided the total number of wasps that made a choice during the test *100 (wasps that did not make a choice were excluded from the calculations of percentage attractiveness). Results of the chemical analysis were analyzed in several ways. Firstly, the compounds emitted by vegetative plants were separated in two categories: leaf compounds and floral compounds. Floral compounds were compounds either only produced or significantly more produced by flowering plants compared to vegetative plants, regardless of infestation. In total, 64 compounds were isolated in *B. rapa* plants: 51 leaf compounds and 13 floral compounds. Secondly, the total volatile emission from plants at each developmental stage before and after infestation by *Pieris* caterpillars was compared for leaf and floral compounds using a paired t test (α = 0.05, JMP9) (n = 11 pairs for both vegetative and flowering plants, n = 12 for pre-flowering plants). Thirdly, the complete blends of volatiles produced by plants at each developmental stage before and after infestation (n = 25 for vegetative plants, n = 24 for pre-flowering plants, n = 23 for flowering plants,) were compared using a principal component analysis (PCA) with the peak areas of the 15 most common compounds found in the blend of vegetative plants, the 25 most common compounds from the blends of pre-flowering plants, and the 27 most common compounds found in the blend of flowering plants. For our analysis of the blends of *B. rapa* plants, we used the two first principal components, accounting for 32 and 19% of the total variation in the dataset for vegetative plants, for 33 and 18% of the total variation in the dataset for pre-flowering plants, and for 22.2 and 16% of the total variation in the dataset for flowering plants. Clear separation between two points on the axes, representing projections of the principal components (Additional file 1: Figure S2), indicates divergence between the whole blends emitted by two plants.

## Results

Overall, the mean percentage of parasitoids that made a choice during the various olfactometer tests was 84.4 ± 3.5 (%, mean ± SE), ranging from 63.2 to 95.3%.

(1) Are pre-flowering and flowering plants infested by *Pieris* caterpillars attractive to parasitoids, and how does this attractiveness compare to infested vegetative plants and non-infested plants?

**Figure 2:**
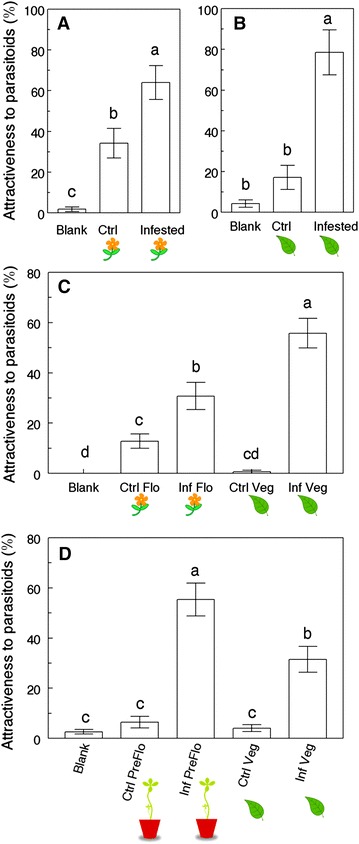
Attractiveness of *Brassica rapa* plants to parasitoids at different developmental stages. Percentage attractiveness of *Cotesia glomerata* females toward different treatments in 4-arm and 6-arm olfactometer settings. Treatments are: *Blank* empty odor source, *Ctrl* non-infested plant, *Infested* or *Inf* *Pieris*-infested plant, *Veg* vegetative plant, *Flo* flowering plant, *PreFlo* plant in the pre-flowering stage. Settings are: **A** 4-arm olfactometer with flowering plants; **B** 4-arm olfactometer with vegetative plants; **C** and **D** 6-arm olfactometers. Treatments followed by a different letter are significantly different (One-way ANOVA, α = 0.05, JMP 9).

Tests conducted in 4-arm olfactometers with vegetative plants and flowering plants separately showed significant differences in attractiveness between the treatments (χ^2^ = 42.2, *P* < 0.0001, and χ^2^ = 49.9, *P* < 0.0001, respectively, df = 3). For both developmental stages, infested plants were more attractive than control plants: infested vegetative plants were five times more attractive than controls, and infested flowering plants twice as attractive as controls (Figure 2A, B). In addition, undamaged flowering plants were more attractive than empty odor sources, while control vegetative plants and empty odor sources had comparably low attractiveness (Figure 2A). When plants from both developmental stages were compared in a 6-arm olfactometer setting, there were significant differences between the treatments (χ^2^ = 103.8, *P* < 0.0001, df = 5): most importantly, infested vegetative plants were significantly more attractive to parasitoids than infested flowering plants (55.8 vs 30.8% attractiveness, respectively) (Figure 2C). On the other hand, when pre-flowering plants were compared to vegetative plants in a 6-arm olfactometer setting, infested pre-flowering plants were 75% more attractive than infested vegetative plants (55.4 vs 31.5% attractiveness, respectively, χ^2^ = 82.1, *P* < 0.0001, df = 3) (Figure 2D).

(2) Do *C. glomerata* show preferences between volatiles emitted by plants infested by its host compared to volatiles emitted by plants damaged by a non-host herbivore, *Spodoptera littoralis*, at different plant developmental stages?

**Figure 3:**
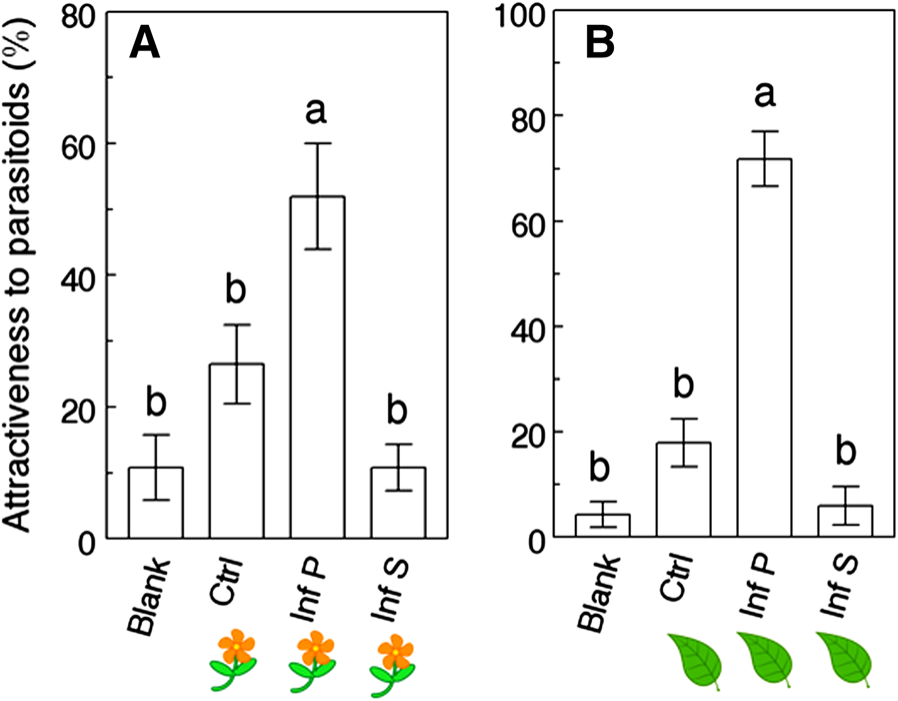
Specificity of plant volatiles emitted by *Brassica rapa* at different developmental stages. Percentage attractiveness of *Cotesia glomerata* females toward different treatments in olfactometer settings. Treatments are: *Blank* empty odor source, *Ctrl* non-infested plant, *Inf* *P* *Pieris*-infested plant, *Inf S* *Spodoptera*-infested plant. Settings are: **A** 4-arm olfactometer with flowering plants; **B** 4-arm olfactometer with vegetative plants. Treatments followed by a different letter are significantly different (One-way ANOVA, α = 0.05, JMP 9).

Tests conducted in 4-arm olfactometers with vegetative plants and flowering plants separately showed significant differences between the treatments (χ^2^ = 59.4, *P* < 0.0001, and χ^2^ = 25.6, *P* < 0.0001, respectively, df = 3). For both developmental stages, the patterns of attractiveness observed were the same: *Pieris*-infested plants were the most attractive treatment, and *Spodoptera*-infested plants were as unattractive as control plants and empty odor sources (Figure 3A, B).

(a) Do floral volatiles directly interfere with attractive HIPVs?

**Figure 4:**
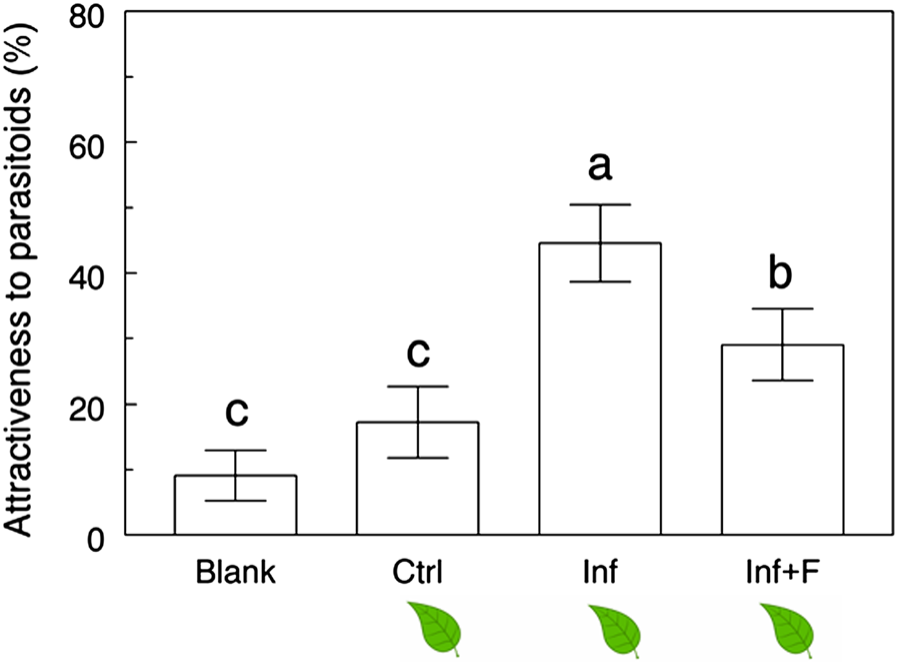
Direct impact of floral odors on parasitoid attraction. Percentage attractiveness of *Cotesia glomerata* females toward different treatments in a 4-arm olfactometer setting. Treatments are: *Blank* empty odor source, *Ctrl* non-infested vegetative plant, *Inf* *Pieris*-infested vegetative plant, *Inf* + *F Pieris*-infested vegetative plant + synthetic floral blend. Treatments followed by a different letter are significantly different (One-way ANOVA, α = 0.05, JMP 9).

The hypothesis that floral volatiles interfere with parasitoid foraging behavior was supported by our results. In a 4-arm olfactometer setting with infested vegetative plants with and without floral odors, we saw the following pattern of preferences (χ^2^ = 23.6, *P* < 0.0001, df = 3): infested plants without synthetic floral blends were the most attractive treatment followed by infested plants with synthetic floral blends, and control plants and empty odor sources were the least attractive treatments. The presence of floral volatiles decreased the attractiveness of infested vegetative plants by approximately 35% (Figure 4).

(b) Is the behavioral change from folivory to florivory in *P. brassicae* caterpillars linked to reduced attractiveness to parasitoids?

**Figure 5:**
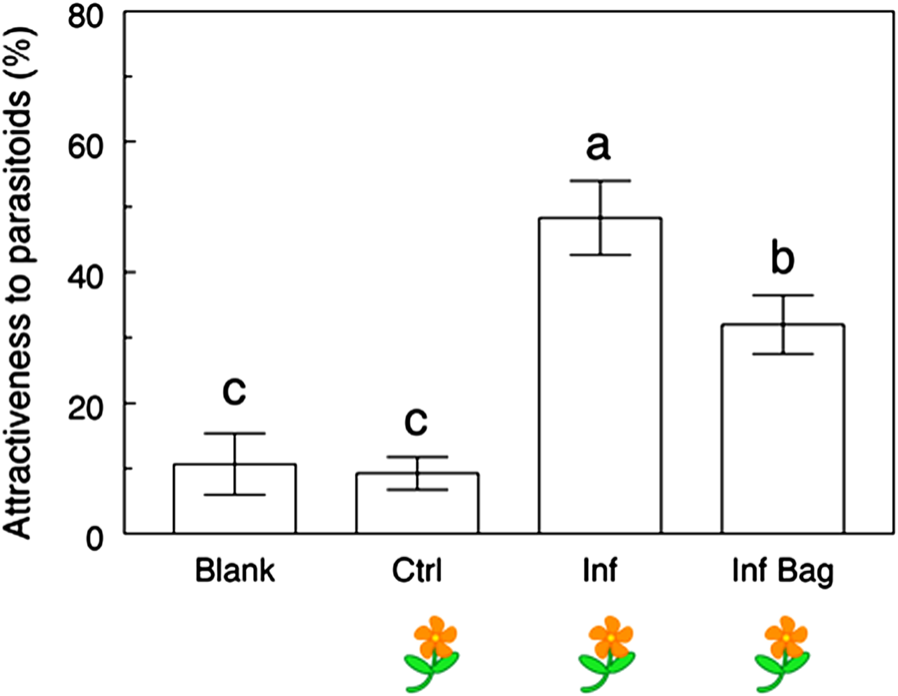
Effect of florivory on parasitoid attraction. Percentage attractiveness of *Cotesia glomerata* females toward different treatments in a 4-arm olfactometer setting. Treatments are: *Blank* empty odor source, *Ctrl* non-infested flowering plant, *Inf Pieris*-infested flowering plant, *Inf Bag* *Pieris*-infested plant whose flowers were bagged to prevent florivory. Treatments followed by a different letter are significantly different (One-way ANOVA, α = 0.05, JMP 9).

The hypothesis that florivory by *P. brassicae* caterpillars reduces the production of attractive HIPVs was not supported by our results. In a 4-arm olfactometer setting with infested flowering plants where caterpillars had access to the flowers or were denied access to the flowers (i.e. flowers were enclosed in bags preventing florivory), the following preferences were observed (χ^2^ = 43.2, *P* < 0.0001): infested plants with access to the flowers were the most attractive treatment, followed by infested plants with enclosed flowers. Infested flowering plants with access to the flowers were 50% more attractive than infested flowering plants with enclosed flowers (48.3 vs. 32.0%, respectively). Both these treatments were more attractive than control plants and empty odor sources (Figure 5).

In all olfactometer tests containing more than one herbivore-infested treatment, the surface of leaf area damaged by herbivores did not significantly differ between treatments (*P*s > 0.05, Additional file 1: Figure S1).

(3) How do herbivore-induced plant volatiles vary through plant ontogeny?

**Figure 6:**
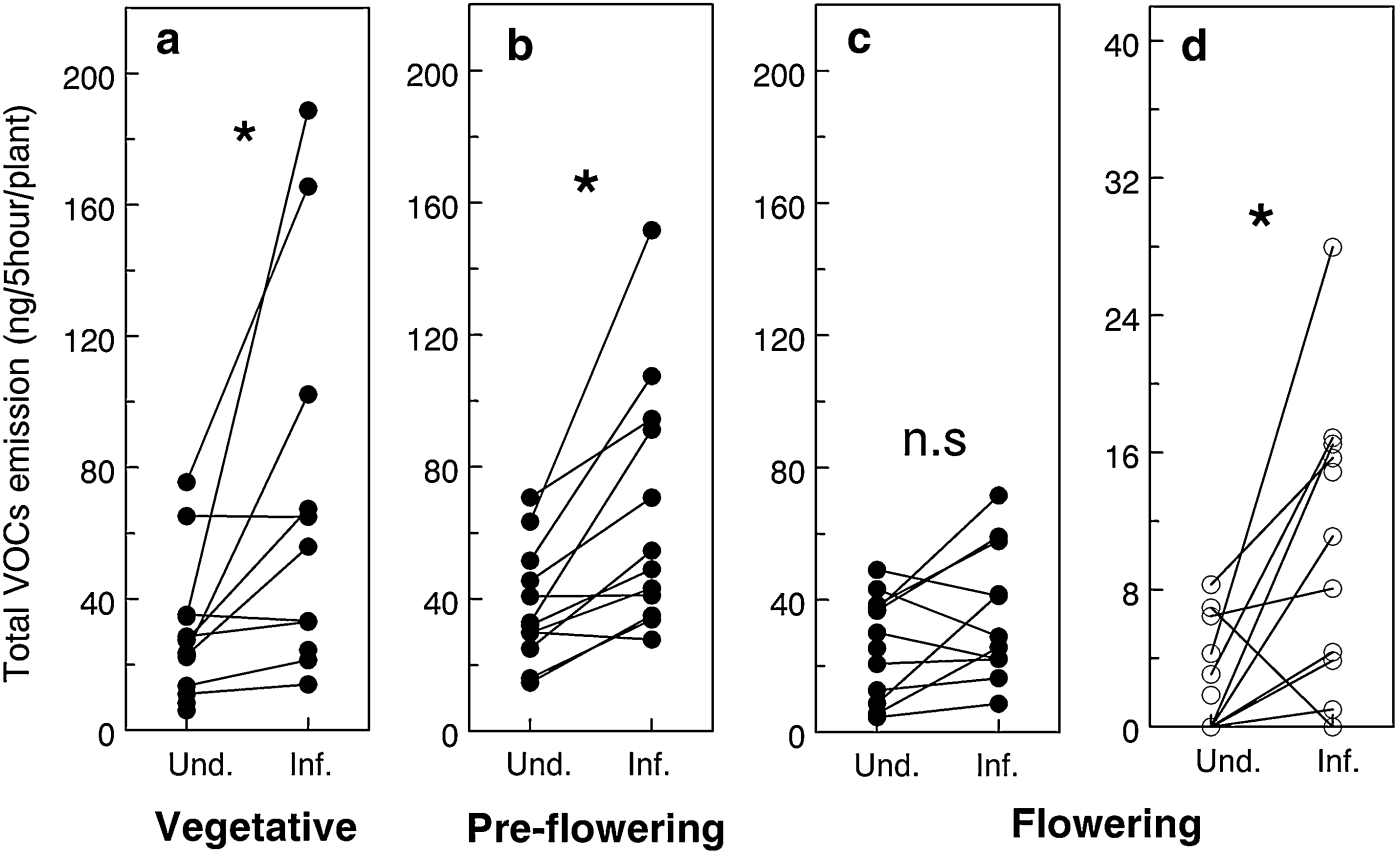
Total volatile emission of vegetative and flowering plants before and after *Pieris* infestation. **a** Leaf volatiles emitted by vegetative plants (n = 11 pairs), **b** leaf volatiles emitted by pre-flowering plants, **c** leaf volatiles emitted by pre-flowering plants, and **d** floral volatiles emitted by flowering plants. Volatiles were collected for 5 h using super Q filters. *Asterisks* indicate significant increase of volatile emission after infestation (paired t test, *P* < 0.05).

Analysis of the complete volatile emission of vegetative, pre-flowering, and flowering plants before and after infestation by *Pieris* caterpillars revealed that the total amount of leaf volatiles emitted was higher after infestation in vegetative and pre-flowering plants (i.e. there was significant induction of these volatiles) (paired t test, t = 2.4, *P* = 0.03 for vegetative plants, and t = 3.83, *P* = 0.003 for pre-flowering plants), and there was a non-significant trend toward induction in flowering plants (t = 1.93, *P* = 0.08). In addition, the total amount of floral volatiles emitted was higher after infestation in flowering plants (t = 3.17, *P* = 0.01) (Figure 6). Principal component analysis of the complete blends of volatiles showed divergence and little overlap between the blends emitted by undamaged and infested plants for each developmental stage tested (Additional file 1: Figure S2).

## Discussion

Plant development and secondary metabolism are tightly linked: the composition and abundance of defensive compounds present in different organs can vary through the lifetime of a single plant. Ontogeny-driven changes in plant metabolism may also affect indirect resistance and attraction of natural enemies [42], through variations in HIPVs [22, 42–44]. Our findings show clearly that parasitoid attraction is ontogeny-dependant in *B. rapa*: compared to infested vegetative plants, infested pre-flowering plants were more attractive and infested flowering plants less attractive to *C. glomerata* females (Figure 2c, d). Considering that the pre-flowering stage is typically only 7–10 days long (from the production of the stalk to the opening of the first flower, GAD personal observation), this result illustrates the highly dynamic nature of the interactions between plants and members of the third trophic level [45, 46]. Independent of developmental stage, infested plants were always considerably more attractive than undamaged plants (Figure 2) and parasitoids were more attracted to plants damaged by their host than to plants damaged by a non-host herbivore, *S. littoralis* (Figure 3). This supports the idea that volatile cues originating from infested *B. rapa* plants remain herbivore-specific during the transition from the vegetative to the reproductive stage, and represent a reliable signal for parasitoids in search of hosts through plant ontogeny, confirming previous results obtained with the same tritrophic system [47]. Because we kept the herbivores on the plants during the bioassays, we cannot dissociate the effects of plant volatiles from the effects of host-derived cues (e.g. frass odors) on parasitoid attraction, and *Pieris*-derived cues may differ from *Spodoptera*-derived cues, playing a role in the specificity of parasitoid attraction. Herbivores damaged comparable amounts of leaf tissue in all treatments (Additional file 1: Figure S1), which suggests that quantity of damage is not responsible for the differences in parasitoid attraction observed during the different experiments.

Analyses of the volatile blends emitted by *B. rapa* plants before and after *Pieris* infestation showed a significant induction of leaf volatiles after infestation in vegetative and pre-flowering plants, but only a trend toward induction in flowering plants (Figure 6), a pattern that is consistent with recent observations in *B. nigra* [43], suggesting that the decreased attractiveness of infested flowering plants is due to a reduced emission of attractive leaf volatiles. There were qualitative differences between the blends of undamaged and *Pieris*-infested plants at all developmental stages, as illustrated by the results of the PCAs (Additional file 1: Figure S2). Taken together with the fact that parasitoids always preferred *Pieris*-infested plants to undamaged plants, these results show that a quantitative induction of leaf volatiles is not necessary to elicit *C. glomerata* attraction, but that the quantity of volatiles induced still plays a role in the strength of this attraction. Intriguingly, induction of leaf volatiles after infestation was more consistent in pre-flowering plants than in vegetative plants. At the vegetative stage, half of the individual plants tested strongly induced leaf volatiles and the other half of plants showing little or null induction, whereas induction was more consistent in pre-flowering plants (80% of plants tested) (Figure 6a, b). This increased consistency in volatile induction may explain the increased attractiveness of infested pre-flowering plants to *C. glomerata* parasitoids.

Regarding floral volatiles, their emission was increased after *Pieris* infestation (Figure 6d) but, because the number of open flowers at the time of volatile collections was not counted, it is possible that the relative amount of floral volatiles released per flower remained stable or even decreased. Because we used plants that started blooming 2–15 days before the volatile collections, an effect of plant age on the odor blends cannot be excluded. Adding synthetic floral scents to infested vegetative plants made them considerably less attractive to parasitoids (Figure 4), which strongly suggests that floral volatiles do in fact negatively interfere with attractive HIPVs in the headspace and contribute to making infested flowering plants less attractive to *C. glomerata* wasps. In this context, caterpillars feeding on or close to the flowers may avoid detection by parasitoids, a theory that is supported by recent work on *B. nigra* [48]. Further experiments are needed to determine to which extent this interference originates from the presence of certain specific compounds in the blend (“qualitative” interference) or if there is a threshold above which the ratio floral/herbivore-induced volatiles becomes detrimental to parasitoid attraction (“quantitative” interference). In parallel, our hypothesis that florivory is linked to decreased attractiveness was not supported by our results (Figure 5). Au contraire, plants with herbivores restrained to the leaves became less attractive than plants with herbivores left free to feed on both leaves and flowers, giving further support to the idea that induction of volatiles is reduced in the leaves of flowering plants.

Natural selection should favor the expression of plant defenses in tissues that are the most valuable or the most at risk of being attacked by herbivores, which may vary through a plant’s lifetime. Reproductive tissues (flowers and seeds) are more valuable than leaf tissue in mature annual plants, as damage inflicted upon them has a direct impact on plant’s fitness [10]. Thus, plants should theoretically benefit from expressing high levels of direct and indirect defense in these tissues: work on *Brassica nigra* showed that flowers indeed contain higher levels of glucosinolates, the main class of defensive compounds in crucifers, than leaves [35]. This pattern is consistent with observations from other systems [10]. Our study, however, indicates that putative indirect resistance traits (herbivore-induced volatiles and attraction of natural enemies) are less effective in flowering *B. rapa* plants. Floral VOCs, which are primarily produced to attract pollinators, seem to inhibit the attraction of parasitoids, indicating a trade-off between pollinator and parasitoid attraction. It is also possible that flowering plants may try to repel parasitoids to avoid pollen and nectar robbery. Work done with the same population of *B. rapa* and the same two herbivores showed that herbivore-infested plants become, after several days of infestation, less attractive to pollinators compared to undamaged plants [41], reinforcing the notion that these two types of plant mutualists (parasitoids and pollinators) are attracted and deterred by different plant volatiles, which may create reciprocal interferences in infochemical networks in nature [49].

The decreased production of herbivore-induced leaf volatiles in flowering *B. rapa* plants may be a direct result of resource constraints and reallocation of resources to reproductive organs. For example, the production of a phytohormone associated with direct defense in the leaves of *Nicotiana attenuata* have been shown to decrease as plants enter the reproductive stage [50]. In our study, plants were constrained by the amount of resources present in the soil of their pots, and reached relatively small sizes at maturity (Figure 1). While this situation is not unrealistic and may correspond to nutrient-poor or competition-rich natural environments, repeating similar experiments with plants less resource-limited would be valuable to test this hypothesis. Alternatively, assuming that attracting natural enemies is beneficial for *B. rapa* plants, producing less leaf VOCs at the flowering stage may have an adaptive value, independently of resource constraints. Because the action of *C. glomerata* is not immediate, it may be too late for infested flowering plants to “cry for help” [30], but more effective to invest in rapid pollination and seed production [51]. However, flowering plants could potentially still benefit from the action of parasitoids killing *P. brassicae* larvae quickly [52], or from the action of generalist predators [53]. The reduced induction of leaf volatiles seems to indicate that flowering plants “broadcast” less cues to foraging natural enemies (although these cues appear to remain herbivore-specific), and that the reduced attraction of *C. glomerata* wasps could be generalized to other types of natural enemies. While the ecological relevance of increased attractiveness of *B. rapa* plants in the pre-flowering stage remains speculative, this result illustrates the highly dynamic nature of the interactions between plants and members of the third trophic level [45, 46].

## Conclusion

In summary, our study constitutes a strong example of rapid, short-term temporal shifts in the preferences of a parasitoid driven by plant ontogeny. In *B. rapa*, attractiveness to parasitoids decreases as plants enter the flowering stage, due to a reduced investment in herbivore-induced volatiles and to the interfering effect of floral volatiles on parasitoid attraction. However, despite this reduced investment in volatiles, infested flowering plants remain more attractive to parasitoids than undamaged plants, and the blend emitted retains its specificity to *Pieris* infestation. Thus, infested flowering *B. rapa* plants may still benefit from attracting natural enemies while reducing the costs of herbivore-induced volatiles on other mutualists (i.e. pollinators) [41]. The negative effects of floral volatiles on parasitoid attraction show that infested flowering plants do not seem to constitute an attractive “double reward” (food + host) in *B. rapa*: further experiments with parasitoids at different levels of food satiation may clarify how parasitoids integrate different volatile clues depending on whether they forage for food or for hosts. In the context of insect–plant interactions, research on the influence of plant ontogeny on herbivory has mostly been focused on the effects of direct defenses on herbivores [10, 11, 54], ignoring members of the third trophic level. By showing that the interactions between plants and natural enemies can rapidly change over a plant’s lifetime, our study underscores the importance of integrating indirect defense when exploring the strategies plants may rely on to face the challenges posed by herbivores over their lifetime.

## Additional files

**Additional file 1**. Total area damaged by caterpillars (cm^2^, mean + SE) in infested plant treatments for the different experiments (Figure S1) and principal component analysis (PCA) of VOCs emitted by undamaged and Pieris-infested *Brassica rapa* plants at each plant developmental stage (Figure S2).
