# Supplementary material for: Floral volatiles interfere with plant attraction of parasitoids: ontogeny-dependent infochemical dynamics in Brassica rapa
Source: BMC Ecol. 2015 Jun 2;15:17. doi: 10.1186/s12898-015-0047-7 (PMC4467598; doi:10.1186/s12898-015-0047-7)
Supplement: Supplementary file 1 — Additional file 1. Total area damaged by caterpillars (cm2, mean + SE) in infested plant treatments for the different experiments (Figure S1) and principal component analysis (PCA) of VOCs emitted by undamaged and Pieris-infested Brassica rapa plants at each plant developmental stage (Figure S2). [file 12898_2015_47_MOESM1_ESM.docx]

**Supporting information**


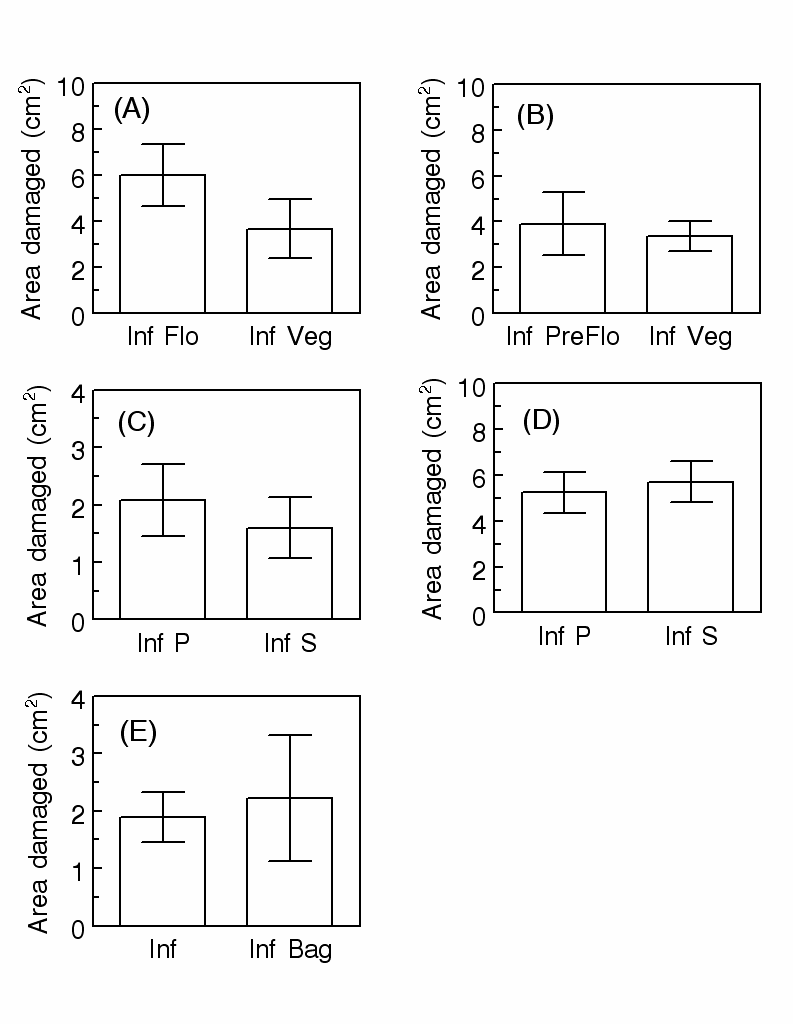


**Figure S1. Total area damaged by caterpillars (cm^2^, mean + SE) in infested plant treatments for the different experiments**. Inf = infested plant, Flo = flowering plant, Veg = vegetative plant, PreFlo = pre-flowering plant, Inf P = *Pieris*-infested plant, S Inf = *Spodoptera*-infested plant, Inf Bag = infested plant whose flowers were bagged to prevent florivory. (A) 6-arm olfactometer with vegetative and flowering plant (Fig. 1C). (B) 6-arm olfactometer with vegetative and pre-flowering plants (Fig. 1D). (C) 4-arm olfactometer with flowering plants (Fig. 4A). (D) 4-arm olfactometer with vegetative plants (Fig. 4B). (E) 4-arm olfactometer with flowering plants and flowers in bags (Fig. 6).


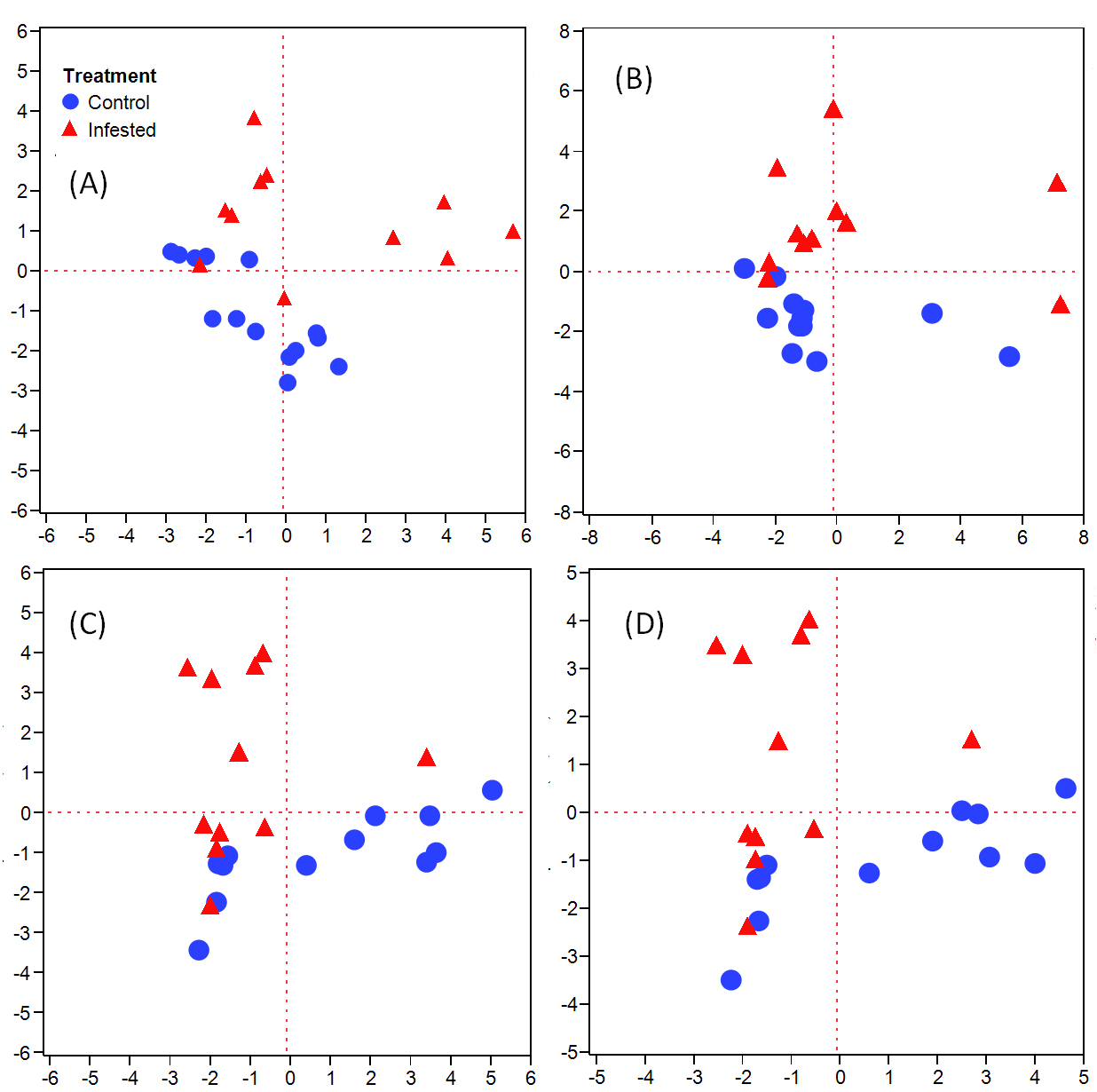


**Figure S2**. **Principal component analysis (PCA) of VOCs emitted by undamaged (blue circles) and *Pieris*-infested (red triangles) *Brassica rapa* plants at the (A) vegetative, (B) pre-flowering, and (C & D) flowering developmental stage**. PCAs of VOCs emitted by flowering plants were performed with (C) both leaf and floral volatiles and (D) leaf volatiles only. The horizontal and vertical axes show projections on to the first and second principal components, respectively. Each dot represents an individual plant (n=25 for vegetative plants, n=24 for pre-flowering plants, and n=23 for flowering plants).
